# Detailed Analysis of Japanese Population Substructure with a Focus on the Southwest Islands of Japan

**DOI:** 10.1371/journal.pone.0035000

**Published:** 2012-04-03

**Authors:** Takeshi Nishiyama, Hirohisa Kishino, Sadao Suzuki, Ryosuke Ando, Hideshi Niimura, Hirokazu Uemura, Mikako Horita, Keizo Ohnaka, Nagato Kuriyama, Haruo Mikami, Naoyuki Takashima, Keitaro Mastuo, Yin Guang, Kenji Wakai, Nobuyuki Hamajima, Hideo Tanaka, for the J-MICC Study Group

**Affiliations:** 1 Clinical Trial Management Center, Nagoya City University Hospital, Nagoya, Japan; 2 Laboratory of Biometry and Bioinformatics, Graduate School of Agriculture and Life Sciences, University of Tokyo, Tokyo, Japan; 3 Department of Public Health, Nagoya City University Graduate School of Medicine, Nagoya, Japan; 4 Department of Nephro-urology, Nagoya City University Graduate School of Medicine, Nagoya, Japan; 5 Department of International Island and Community Medicine, Kagoshima University Graduate School of Medical and Dental Sciences, Kagoshima, Japan; 6 Department of Preventive Medicine, Institute of Health Biosciences the University of Tokushima Graduate School, Tokushima, Japan; 7 Department of Preventive Medicine, Faculty of Medicine, Saga University, Saga, Japan; 8 Department of Geriatric Medicine, Graduate School of Medical Sciences, Kyushu University, Fukuoka, Japan; 9 Department of Epidemiology for Community Health and Medicine, Kyoto Prefectural University of Medicine Graduate School of Medical Science, Kyoto, Japan; 10 Division of Cancer Registry, Prevention and Epidemiology, Chiba Cancer Center, Chiba, Japan; 11 Department of Health Science, Shiga University of Medical Science, Otsu, Japan; 12 Division of Epidemiology and Prevention, Aichi Cancer Center Research Institute, Nagoya, Japan; 13 Department of Preventive Medicine, Nagoya University Graduate School of Medicine, Nagoya, Japan; Biodiversity Insitute of Ontario - University of Guelph, Canada

## Abstract

Uncovering population structure is important for properly conducting association studies and for examining the demographic history of a population. Here, we examined the Japanese population substructure using data from the Japan Multi-Institutional Collaborative Cohort (J-MICC), which covers all but the northern region of Japan. Using 222 autosomal loci from 4502 subjects, we investigated population substructure by estimating F_ST_ among populations, testing population differentiation, and performing principal component analysis (PCA) and correspondence analysis (CA). All analyses revealed a low but significant differentiation between the Amami Islanders and the mainland Japanese population. Furthermore, we examined the genetic differentiation between the mainland population, Amami Islanders and Okinawa Islanders using six loci included in both the Pan-Asian SNP (PASNP) consortium data and the J-MICC data. This analysis revealed that the Amami and Okinawa Islanders were differentiated from the mainland population. In conclusion, we revealed a low but significant level of genetic differentiation between the mainland population and populations in or to the south of the Amami Islands, although genetic variation between both populations might be clinal. Therefore, the possibility of population stratification must be considered when enrolling the islander population of this area, such as in the J-MICC study.

## Introduction

Uncovering population structure is a crucial step in properly conducting association studies because neglecting to correct for population structure can lead to both false positive results and failures to detect genuine associations [1]–[3]. An understanding of the population structure is also important in population genetics, especially to uncover the demographic history of a population under study [4].

**Figure 1:**
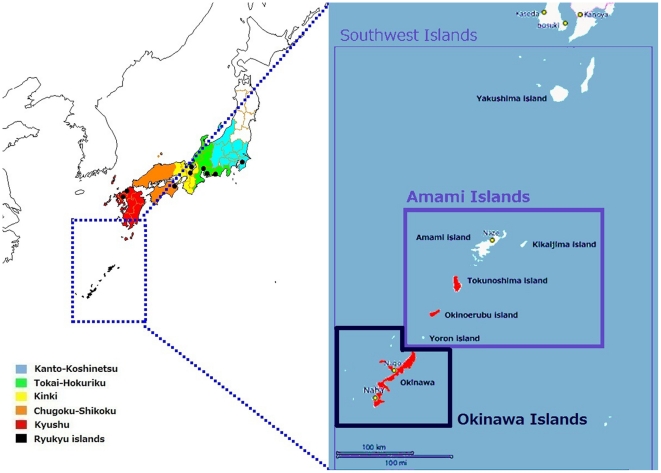
Geographic locations of the populations studied in Japan. Kanto-Koshinetsu: the eastern-central region of the main island. Tokai-Hokuriku: the central region of the main island. Kinki: the southern-central region of the main island. Chugoku-Shikoku: the westernmost part of the main island and the fourth largest island. Kyushu: the third largest island, located southwest of the main island. The Amami Islands: a part of the Southwest Islands, located southwest of Kyushu. The black circles represent the approximate geographic positions of the enrollment institutions, and the red-colored islands in the enlarged view of Japan's Southwest Islands (right) represent those used for sampling in the J-MICC study (Tokunoshima and Okinoerabu Islands) and in the survey by the PASNP consortium (the Okinawa Islands).

It is generally accepted that the modern Japanese population was formed by the mixture of two major ancestral groups who came to Japan by different routes at different times. The mainland population of Japan shows genetic influences from both groups but appears to be predominantly descended from the second ancestral group, whereas two contemporary indigenous groups in Japan, the Ainu and Ryukyu peoples, are recognized as remnant populations descended from the first ancestral group [5]–[10]. These peoples inhabit both ends of the Japanese archipelago: the Ainu people live on the northern island of Hokkaido, and the Ryuku people live on the southernmost islands, called Japan's Southwest Islands, including the Okinawa Islands (Figure 1).

A previous study of the Japanese population substructure, based on genome-wide association study (GWAS) data, revealed the clear differentiation between the Ryukyu and mainland populations and partially confirmed the dual structure hypothesis described above [11]. However, genetic differentiation has not been well examined along the Southwest Islands between Okinawa Islands and the Kyushu (Japan's southernmost mainland). Previous studies [5]–[10] used only the inhabitants of Okinawa Island, the geographical and political center of the Southwest Islands (Figure 1), as a representative sample of the Ryukyu population. Here, we focused on the Amami Islands, located midway between Okinawa Island and Kyushu. The proponent of the dual structure hypothesis suggests that the Amami Islanders should be included in the Ryukyu population (Figure 1) [5]; however, a detailed analysis of the Amami Islanders has not yet been conducted.

Therefore, the aim of this study is to examine the genetic differentiation between the Amami Islanders and other Japanese subpopulations (Figure 1). For this purpose, we used data from the Japan Multi-Institutional Collaborative Cohort (J-MICC), which was launched in 2005 to detect gene-environment interactions in the development of life style-related diseases, particularly cancer. This study enrolled subjects in ten study areas throughout Japan (except the northern region), including the Amami Islands (Figure 1) [12], [13]. If a large differentiation between the Amami Islands and the other areas in Japan is observed, correction for population stratification is required in association studies that use a sample including the Amami Islanders, such as the J-MICC study.

## Materials and Methods

### Subjects

Genotype data were obtained from 4514 Japanese subjects in the J-MICC study, which is one of the largest population-based cohort studies in Japan [12], [13]. The subjects were enrolled in ten study areas throughout Japan, except the northern region.Although information on geographic locations of the sampled individuals was not available in this study, the approximate geographic positions of the enrollment institutions are shown in Figure 1: 506 subjects were enrolled in Kanto-Koshinetsu (the eastern-central region of the main island), 1676 in Tokai-Hokuriku (the central region of the main island), 702 in Kinki (the southern-central region of the main island), 95 in Chugoku-Shikoku (the westernmost part of the main island and the fourth largest island), 1020 in Kyushu (the third largest island, located southwest of the main island), and 515 in the Amami Islands (part of the Southwest Islands, located southwest of Kyushu). We note that the Amami Islanders were sampled from the Tokunoshima and Okinoerabu Islands (Figure 1). Throughout this paper, we refer to the four largest main islands of Japan (Hokkaido, Honshu, Shikoku, and Kyushu) as “the mainland”.

In addition to the J-MICC data, the Pan-Asian SNP (PASNP) consortium genotype data [14] were also used so that samples from the Okinawa Islands could be included in this study. The genotype data for 49 Okinawa Islanders and 71 mainland individuals were selected from the PASNP database.

The ethics committees of all participating institutions approved the protocol for the J-MICC study, and all participants provided written informed consent.

The participating institutions included:

1. Division of Cancer Registry, Prevention and Epidemiology, Chiba Cancer Center, Chiba, Japan,
2. Department of Preventive Medicine, Nagoya University Graduate School of Medicine, Nagoya, Japan
3. Division of Epidemiology and Prevention, Aichi Cancer Center Research Institute, Nagoya, Japan,
4. Department of Public Health, Nagoya City University Graduate School of Medicine, Nagoya, Japan,
5. Department of Health Science, Shiga University of Medical Science, Otsu, Japan,
6. Department of Epidemiology for Community Health and Medicine, Kyoto, Japan,
7. Prefectural University of Medicine Graduate School of Medical Science, Kyoto, Japan,
8. Department of Preventive Medicine, Institute of Health Biosciences, University of Tokushima Graduate School, Tokushima, Japan,
9. Department of Preventive Medicine, Graduate School of Medical Sciences, Kyushu University, Fukuoka, Japan,
10. Department of Preventive Medicine, Faculty of Medicine, Saga University, Saga, Japan,
11. Department of International Island and Community Medicine, Kagoshima University Graduate School of Medical and Dental Sciences, Kagoshima, Japan and
12. Laboratory for Genotyping Development, Center for Genomic Medicine, RIKEN

### Genotyping

**The J-MICC data:** All genotypes were determined using the multiplex PCR-based Invader assay (Third Wave Technologies, Madison, WI, USA) at the Laboratory for Genotyping Development, Center for Genomic Medicine, RIKEN [15].

In this study, we used 303 SNPs and one insertion/deletion (indel) originally designed for association studies [12], [13]. Among the initial 303 SNPs and 1 indel used, monomorphic polymorphisms (19 SNPs), polymorphisms with extreme deviation from HWE (p<0.00001; 6 SNPs), polymorphisms with a minor allele frequency (MAF)<1% (1 SNP) and polymorphisms that were in linkage disequilibrium with each other (r^2^>0.5; 52SNPs) were excluded from the data set. The remaining polymorphisms had call rates of >90% and were not excluded. Subjects with a call rate less than 90% (12 subjects) were excluded. Thus, the final data set for further analyses consisted of 221 autosomal SNPs and one autosomal indel for 4502 subjects (Table S1).

**The PASNP data:** We also used all 54794 autosomal SNP genotypes available in the PASNP data. After applying the same filtering procedures used for the J-MICC data to the PASNP data, 46485 SNPs remained. Of these SNPs, only six (rs1154460, rs10516441, rs3897749, rs1342382, rs10492024 and rs936306) were contained in the J-MICC data; thus, these SNPs were used for comparison between populations of the mainland, Amami Islands and Okinawa Islands.

Data filtering, the calculation of basic summary statistics, and Hardy-Weinberg equilibrium (HWE) tests were performed with the R package SNPassoc [16].

### Analysis

To measure the differentiation between populations, the widely used statistic F_ST_ [17] and its unbiased estimator [18] were used. F_ST_ estimates were averaged over all loci, and 95% confidence intervals (CIs) of the average F_ST_ were calculated by bootstrap resampling with 10000 replications. We used this computational method so that this study would be comparable with that of Yamaguchi-Kabata *et al.* [11]. The F_ST_ over all loci was also estimated as the ratio of sums of the variance components in the numerator and denominator [19]. Along with F_ST_, variance components were estimated to reflect intra-individual, inter-individual and inter-population differences in genetic variation.

To test for differentiation between two populations based on multiple loci, Goudet's G statistic was used with 10000 permutations of individuals between populations [20].The Cochran-Mantel-Haenszel test was used to test for differences in the levels of heterozygosity across multiple loci between two populations. This test compares the proportions of heterozygotes at each locus between two populations across the strata of loci.

A principal component analysis (PCA) was performed to visualize the relationship between populations; this type of analysis summarizes information for multiple loci into a few synthetic variables called *principal components*.

For the same purpose, correspondence analysis (CA) was performed on a contingency table of minor allele counts per population. Importantly, as in any analysis carried out at a population level, all information about the diversity within populations is lost in this analysis. In contrast, PCA was performed at the individual level and not at the population level.

F_ST_ estimations, variance component estimations and tests of population differentiation at multiple loci were all performed with the R package hierfstat [21]. Both PCA and CA were performed with the R package adegenet [22]. All analyses, except as otherwise noted, were performed using R version 2.9.0 for Windows [23].

## Results

The average F_ST_ over all loci and its 95% CI between each pair of subpopulations in the J-MICC data are shown in Table 1. The F_ST_ values between the Amami Islanders and other mainland Japanese subpopulations (0.0067–0.0086) were much larger than the F_ST_ values between mainland Japanese subpopulations (0.0001–0.0007).

**Table 1:** Genetic differentiation among subpopulations in the J-MICC data.

	Tokai-Hokuriku	Kinki	Chugoku-Shikoku	Kyushu	Amami Islands
Kanto-Koshinetsu	0.0002	0.0001	0.0007	0.0001	0.0073
	(0.0000, 0.0004)	(−0.0001, 0.0003)	(0.0001, 0.0014)	(0.0000, 0.0003)	(0.0059, 0.0088)
Tokai-Hokuriku		0.0002	0.0003	0.0001	0.0076
		(0.0001, 0.0003)	(−0.0002, 0.0009)	(0.0000, 0.0003)	(0.0062, 0.0092)
Kinki			0.0006	0.0003	0.0082
			(−0.0001, 0.0013)	(0.0001, 0.0005)	(0.0066, 0.0100)
Chugoku-Shikoku				0.0005	0.0086
				(0.0000, 0.0010)	(0.0064, 0.0110)
Kyushu					0.0067
					(0.0053, 0.0082)

F_ST_ values were averaged over 222 autosomal loci (221 SNPs and one indel), and 95% confidence intervals were computed using 10000 bootstrap resamplings.

To further clarify the differences between the Amami Islanders and the mainland subpopulations, we estimated the F_ST_ values for all loci; the resulting distribution is shown in Figure S1. First, we examined the average F_ST_ and its 95% CI between the Amami Islanders and a population grouped across all subpopulations in the mainland. Once again, a substantially large F_ST_ value was observed (0.0075, 95%CI: 0.0060–0.0091). Furthermore, this genetic differentiation was statistically significant according to the G statistic (p<0.001) [20]. Similar results were found when F_ST_ was estimated as the combined ratio estimate over all loci (Table S2).

A variance component analysis of the J-MICC data revealed that variations between the Amami Islanders and the mainland population and among individuals within each of both groups explained 0.8% and 0.7% of the total variation, respectively and most of the genetic variation was contained within individuals (Table 2). When the genetic diversity within each group was examined by means of mean heterozygosity across all loci, the mainland population presented a significantly smaller mean heterozygosity (≈0.0865) than the Amami Islanders (≈0.0953, p<0.0001, Figure S2), despite the lack of clear substructure within the mainland.

**Table 2:** Variance components for the J-MICC data.

		Among subpopulations	Among individuals within subpopulations	Within individuals
Amami vs. Mainland	variance components (95% CI)	0.64 (0.51, 0.77)	0.59 (0.40, 0.75)	78.13 (74.10, 82.06)
	relative proportion (%)	0.8%	0.7%	98.5%

The total genetic variation is partitioned into variations between two subpopulations (“Among subpopulations”), among individuals within each subpopulation (“Among individuals within subpopulations”) and within individuals. The relative proportions (%) and 95% confidence intervals (95% CI) for variance components are also shown.

**Figure 2:**
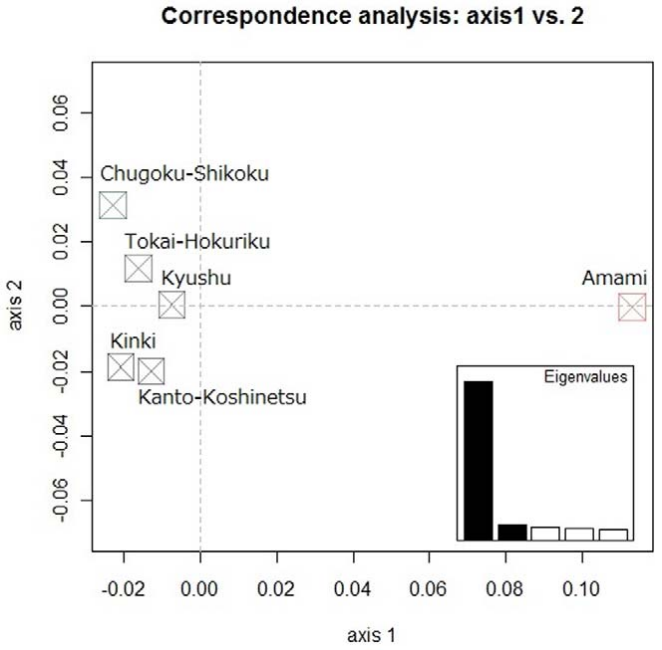
Correspondence analysis plot of the first and second principal components for all subpopulations in the J-MICC. Correspondence analysis was conducted using 222 loci for six subpopulations (Kanto-Koshinetsu, Tokai-Hokuriku, Kinki, Chugoku-Shikoku, Kyushu and the Amami Islands) in the J-MCC. The scree plot is shown in the lower right corner of this figure.

Although PCA did not clearly separate the Amami Islanders from the other subpopulations of the mainland (Figure S3, S4, and S5), CA did clearly separate the Amami Islanders from the other subpopulations (Figure 2). As shown in the scree plot in the lower right portion of Figure 2, the first principal component accounts for a vast majority (≈75%) of the variability; the separation between the Amami Islanders and the rest of the subpopulations was described by this key principal axis.

Next, we examined the genetic differentiation between the mainland population, Amami Islanders and Okinawa Islanders using the same methods used for the J-MICC data. The average F_ST_ over the six SNPs that were contained in both the J-MICC data and the PASNP data and its 95% CI between each pair of subpopulations are shown in Table 3 and Table S3. Notably, the Okinawa Islanders are slightly more genetically differentiated from the mainland population (F_ST_ = 0.0125, p = 0.03) than the Amami Islanders are (F_ST_ = 0.0087, p<0.001), and both Islanders groups are similar to each other (F_ST_ = −0.0003, p = 0.453). The difference in the p-values for comparisons of each Islanders group with the mainland population reflects the different sample sizes of the Islander populations (only 49 Okinawa Islanders vs. 515 Amami Islanders).

**Table 3:** Genetic differentiation between the mainland population, Amami Islanders and Okinawa Islanders.

	Amami Islands	Okinawa Islands (PASNP)
Mainland	0.0087	0.0125
	(0.0014, 0.0170)	(−0.0050, 0.0396)
Amami Islands		−0.0003
		(−0.0056, 0.0076)

F_ST_ values were averaged over six SNPs and 95% confidence intervals were computed using 10000 bootstrap resamplings. The mainland population is grouped across all subpopulations in the mainland, *i.e.*, Kanto-Koshinetsu, Tokai-Hokuriku, Kinki, Chugoku-Shikoku, and Kyushu. Genotype data of the Okinawa Islanders were obtained from the Pan-Asian SNP (PASNP) consortium database.

**Figure 3:**
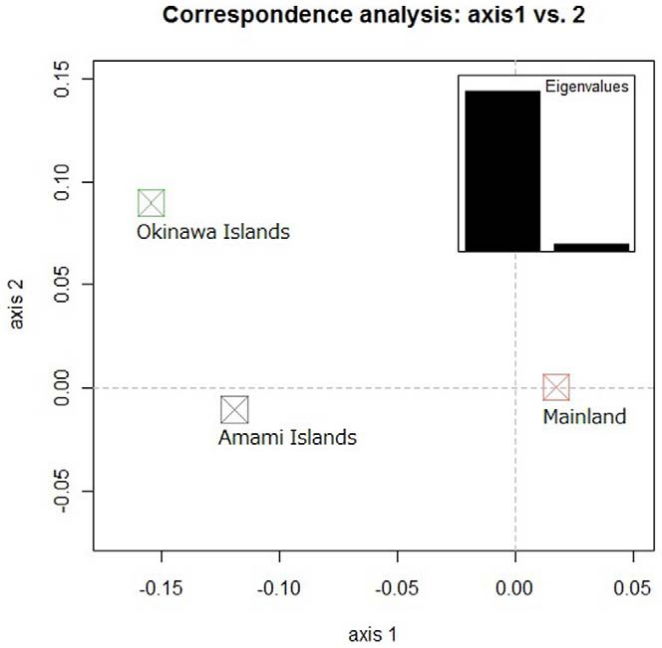
Correspondence analysis plot of the first and second principal components for the mainland population, Amami Islanders and Okinawa Islanders. Correspondence analysis for the mainland population, Amami Islanders and Okinawa Islanders was conducted using six loci. The scree plot is shown in the upper right corner of this figure.

The variance component between both Islanders is 0.2%, which is about one order of magnitude less than that between the mainland population and either Islanders group (1.2% for the Amami Islanders and 1.9% for the Okinawa Islanders; Table 2 and Table 4). In the CA plot for the first and second principal components, the Okinawa Islanders are also slightly more distant from the mainland population than the Amami Islanders are (Figure 3). That is, the CA result is generally consistent with the pattern suggested by the relative paired F_ST_ values with respect to the distance separation among the three groups.

**Table 4:** Variance components for the J-MICC and the PASNP data.

		Among subpopulations	Among individuals within subpopulations	Within individuals
Amami vs. Mainland	variance components (95% CI)	0.03 (0.00, 0.05)	0.02 (0.00, 0.06)	2.13 (1.42, 2.74)
	relative proportion (%)	1.2%	1.1%	97.7%
Okinawa vs. Mainland	variance components (95% CI)	0.04 (−0.01, 0.13)	0.03 (0.00, 0.07)	2.12 (1.48, 2.74)
	relative proportion (%)	1.9%	1.4%	96.7%
Amami vs. Okinawa	variance components (95% CI)	<0.01 (−0.01, 0.03)	<0.01 (−0.09, 0.06)	2.20 (1.45, 2.85)
	relative proportion (%)	0.2%	0.2%	99.6%

The total genetic variation is partitioned into variations between two subpopulations (“Among subpopulations”), among individuals within each subpopulation (“Among individuals within subpopulations”) and within individuals. The relative proportions (%) and 95% confidence intervals (95% CI) for variance components are also shown.

## Discussion

Our study has clearly shown that both the Amami and Okinawa Islanders are genetically differentiated from the mainland Japanese population. Because a differentiation between the Ryukyu and mainland population has also been demonstrated [11], the Amami Islanders are suggested to belong predominantly to the Ryukyu population. Previous dental morphological studies found that the modern inhabitants of Tanegashima Island, just south of Kyushu, are most similar to the mainland Japanese (Figure 1) [24], [25]. Thus, we suggest a genetic boundary between the Amami Islands and Tanegashima Island, which should be further verified (Figure 1).

In the presence of the population structure observed here, a high incidence of false positives may be observed in association studies. This problem arises because allele frequencies can differ between the Amami and mainland population, and also the two population frequencies can differ between case and control groups. For the sample size required for the study of complex diseases, relatively modest levels of structure within a population can have serious consequences [3]. Therefore, population structure cannot be safely ignored in association studies that use a structured population, such as in the J-MICC study.

In this study, a low but significant F_ST_ value was observed between the Amami and the mainland populations; this value was similar to the F_ST_ value between the mainland Japanese population and the Han Chinese in Beijing (CHB) [26]. However, this F_ST_ value (≈0.008) is slightly larger than that obtained in a previous study of the Japanese population substructure based on GWAS data (≈0.003) [11]. This result may be due to the different allelic spectra between polymorphisms in our study and those in the previous study; the former uses polymorphisms originally designed for candidate association studies, whereas the latter uses those designed for GWAS.

Although we can separate the Amami Islanders and the mainland population using correspondence analysis (CA), we cannot separate the two groups using principal component analysis (PCA) because the number of polymorphisms (222 loci) used in this study is not large enough to classify individuals according to the two subpopulations. According to the study that first proposed regression to the principal components of the PCA to correct for population stratification [27], sample size does not affect the accuracy of assigning individuals into subgroups. However, the number of SNPs used to infer population structure greatly affects accuracy. In fact, when we conducted a PCA for 49 Okinawa Islanders and 71 mainland individuals in the PASNP data using all 46485 SNPs and then using 5000, 1000, 900 and 800 SNPs that were randomly selected from all of the SNPs (Figure S5), the separation between the two groups became less clear as the number of SNPs decreased, and no separation was found at 800 SNPs. This result clearly demonstrates that the number of polymorphisms (222 loci) used in our study is too small to separate the Amami Islanders and the mainland population using PCA at an individual level. In contrast, CA at the population level (even though CA can be applied to individual-level data by context) was able to detect the population substructure in our sample because our data have sufficient information to detect the substructure at the resolution of the population (but not the individual) level.

Finally, it should be noted that the low coverage of study areas in this study might exaggerate the sharpness of the observed genetic boundary between the mainland population and the Amami Islanders, although the true pattern of genetic variation might be clinal. Therefore, we think that it is necessary for further studies to include individuals from the southernmost mainland part of Japan (southern Kyushu) and a few other islands between the Amami Islands and the mainland.

In conclusion, we have revealed a low but significant level of genetic differentiation between the mainland population and population in or to the south of the Amami Islands, including the Okinawa Islands, although the genetic variation between both populations might be clinal. Therefore, the possibility of population stratification must be considered when enrolling the islander population of this area, such as in the J-MICC study.

## Supporting Information

Figure S1 **Empirical distribution of F_ST_ values per locusbetween the Amami Islanders and the mainland population (JPEG).**
(TIF)

Figure S2 **Empirical distribution of heterozygosity per locus for the Amami Islanders and the mainland population (JPEG).**
(TIF)

Figure S3 **Scree plot of principal component analysis for the mainland population and the Amami Islanders in the J-MICC (JPEG).**
(TIF)

Figure S4 **Principal component analysis plot for the mainland population and the Amami Islanders in the J-MICC.** (a) PCA plot of the first and second principal components, (b) PCA plot of the first and third principal components (JPEG).
(TIF)

Figure S5 **Principal component analysis plot of the first and second principal components for the mainland population and the Okinawa Islanders in the PASNP data.** Principal component analysis was conducted for the mainland population and the Okinawa Islanders in the PASNP data, using (a) all 46485 loci and (b) 5000, (c) 1000, (d) 900 and (e) 800 loci that were randomly selected from all loci. Scree plots are shown in each figure.
(TIF)

Table S1 (DOC)

Table S2 (DOC)

Table S3 (DOC)
