# Supplementary figures and images for: Detailed Analysis of Japanese Population Substructure with a Focus on the Southwest Islands of Japan
Source: PLoS One. 2012 Apr 3;7(4):e35000. doi: 10.1371/journal.pone.0035000 (PMC3318002; doi:10.1371/journal.pone.0035000)

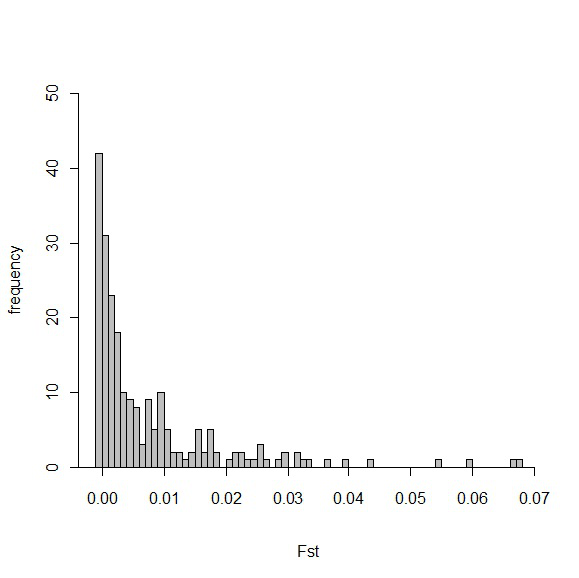

Supplement: Figure S1 — Empirical distribution of FST values per locusbetween the Amami Islanders and the mainland population (JPEG). (TIF) [file pone.0035000.s001.tif]

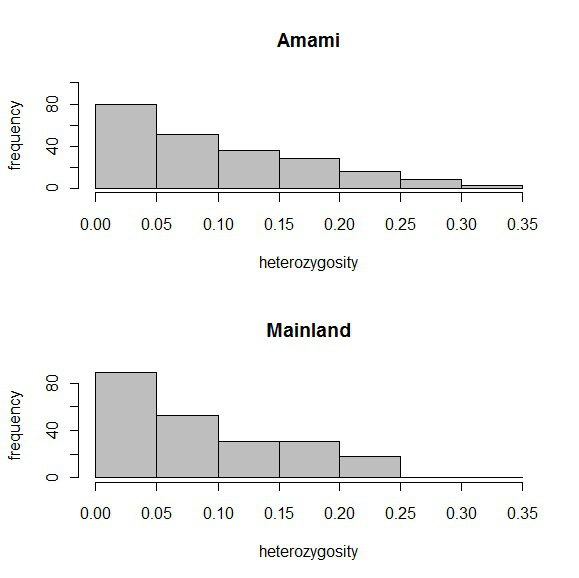

Supplement: Figure S2 — Empirical distribution of heterozygosity per locus for the Amami Islanders and the mainland population (JPEG). (TIF) [file pone.0035000.s002.tif]

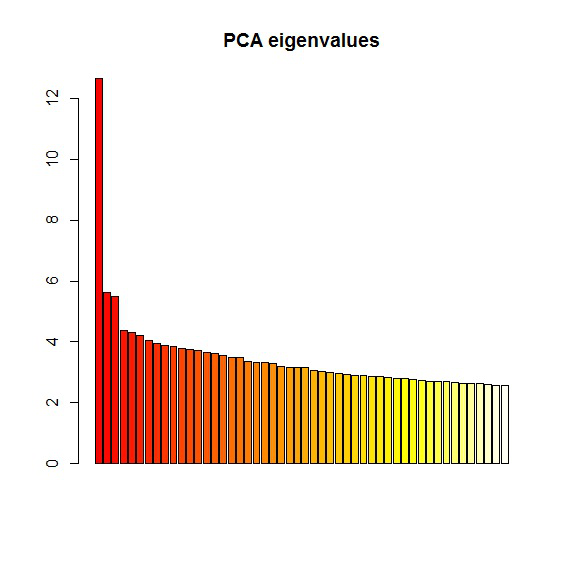

Supplement: Figure S3 — Scree plot of principal component analysis for the mainland population and the Amami Islanders in the J-MICC (JPEG). (TIF) [file pone.0035000.s003.tif]

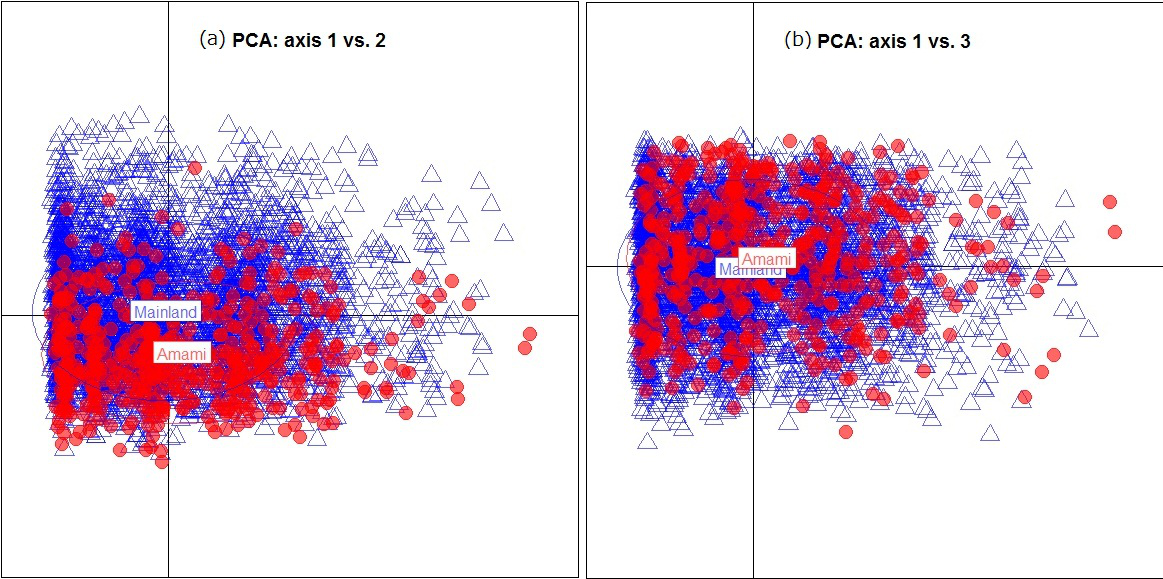

Supplement: Figure S4 — Principal component analysis plot for the mainland population and the Amami Islanders in the J-MICC. (a) PCA plot of the first and second principal components, (b) PCA plot of the first and third principal components (JPEG). (TIF) [file pone.0035000.s004.tif]

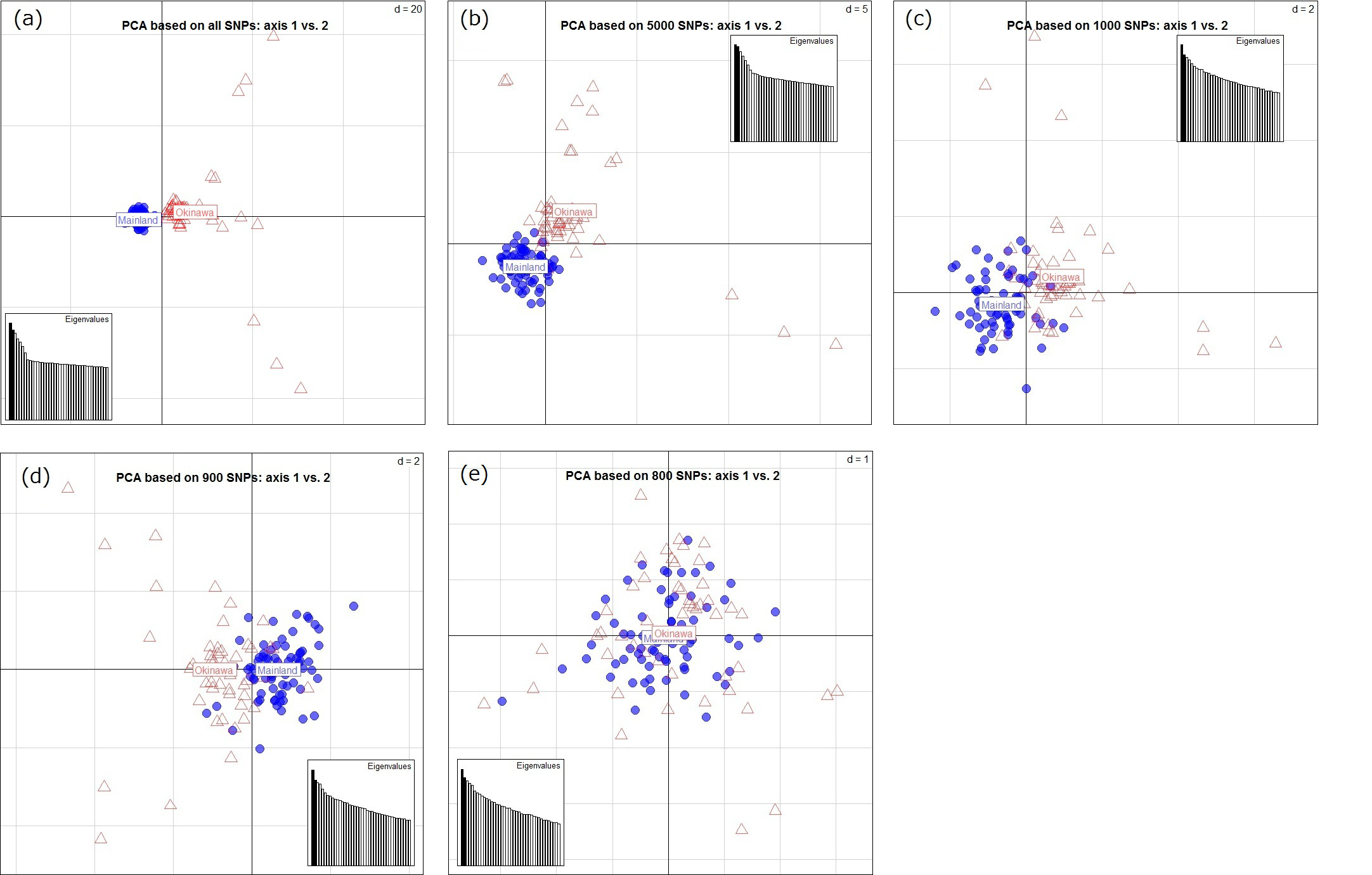

Supplement: Figure S5 — Principal component analysis plot of the first and second principal components for the mainland population and the Okinawa Islanders in the PASNP data. Principal component analysis was conducted for the mainland population and the Okinawa Islanders in the PASNP data, using (a) all 46485 loci and (b) 5000, (c) 1000, (d) 900 and (e) 800 loci that were randomly selected from all loci. Scree plots are shown in each figure. (TIF) [file pone.0035000.s005.tif]
