# Supplementary material for: Detailed Analysis of Japanese Population Substructure with a Focus on the Southwest Islands of Japan
Source: PLoS One. 2012 Apr 3;7(4):e35000. doi: 10.1371/journal.pone.0035000 (PMC3318002; doi:10.1371/journal.pone.0035000)
Supplement: Table S1 — (DOC) [file pone.0035000.s006.doc]

**Table S1**

**Description of polymorphisms used in the J-MCC**

| name | chr | pos | gene |
| --- | --- | --- | --- |
| rs1801131 | 1 | 11854476 | MTHFR |
| rs1801133 | 1 | 11856378 | MTHFR |
| rs7414562 | 1 | 14359780 | PRDM2 |
| rs10903129 | 1 | 25768937 | TMEM57 |
| rs3219489 | 1 | 45797505 | MUTYH |
| rs11206510 | 1 | 55496039 | PCSK9 |
| rs1418442 | 1 | 57159136 | PRKAA2 |
| rs1342382 | 1 | 57177388 | PRKAA2 |
| rs1167998 | 1 | 62931632 | DOCK7 |
| rs6698181 | 1 | 89143305 | PKN2 |
| rs11165976 | 1 | 98755006 | LOC729987 |
| rs12129861 | 1 | 145725689 | PDZK1 |
| rs2072658 | 1 | 154540225 | CHRNB2 |
| rs2072660 | 1 | 154548721 | CHRNB2 |
| rs3737787 | 1 | 161009523 | TSTD1,USF1 |
| rs677 | 1 | 173876561 | SERPINC1 |
| rs5270 | 1 | 186649719 | PTGS2 |
| rs20417 | 1 | 186650321 | PTGS2 |
| rs7539542 | 1 | 202909974 | ADIPOR1 |
| rs1342387 | 1 | 202914356 | ADIPOR1 |
| rs1800871 | 1 | 206946634 | IL10 |
| rs699 | 1 | 230845794 | AGT |
| rs4762 | 1 | 230845977 | AGT |
| rs1805087 | 1 | 237048500 | MTR |
| rs12564791 | 1 | 247594890 | NLRP3 |
| rs673548 | 2 | 21237544 | APOB |
| rs17042603 | 2 | 21798418 | unknown |
| rs1260326 | 2 | 27730940 | GCKR |
| rs523349 | 2 | 31805706 | SRD5A2 |
| rs1143627 | 2 | 113594387 | IL1B |
| rs6436094 | 2 | 219687597 | PRKAG3 |
| rs692243 | 2 | 219695487 | PRKAG3 |
| rs934945 | 2 | 239155053 | PER2 |
| rs1052133 | 3 | 9798773 | CAMK1,OGG1 |
| rs1801282 | 3 | 12393125 | PPARG |
| rs3856806 | 3 | 12475557 | PPARG |
| rs5186 | 3 | 148459988 | AGTR1 |
| rs4402960 | 3 | 185511687 | IGF2BP2 |
| rs266729 | 3 | 186559474 | ADIPOQ |
| rs822396 | 3 | 186566877 | ADIPOQ |
| rs2241766 | 3 | 186570892 | ADIPOQ |
| rs1501299 | 3 | 186571123 | ADIPOQ |
| rs4961 | 4 | 2906707 | ADD1 |
| rs1014290 | 4 | 10001861 | SLC2A9 |
| rs8192678 | 4 | 23815662 | PPARGC1A |
| rs2970847 | 4 | 23815924 | PPARGC1A |
| rs1801260 | 4 | 56301369 | CLOCK |
| rs3736544 | 4 | 56309992 | CLOCK |
| rs4864548 | 4 | 56413803 | CLOCK |
| rs4073 | 4 | 74606024 | IL8 |
| rs2231142 | 4 | 89052323 | ABCG2 |
| rs2602836 | 4 | 100014805 | unknown |
| rs4148887 | 4 | 100055862 | ADH4 |
| rs3805322 | 4 | 100056998 | ADH4 |
| rs4147547 | 4 | 100127311 | ADH6 |
| rs3819197 | 4 | 100200509 | ADH1A |
| rs4147532 | 4 | 100211786 | ADH1A |
| rs13103321 | 4 | 100220240 | LOC100288807 |
| rs1229984 | 4 | 100239319 | ADH1B |
| rs1229982 | 4 | 100243932 | ADH1B |
| rs4147541 | 4 | 100274157 | ADH1C |
| rs284787 | 4 | 100333556 | ADH7 |
| rs284785 | 4 | 100335586 | ADH7 |
| rs1154456 | 4 | 100339596 | ADH7 |
| rs1154460 | 4 | 100341643 | ADH7 |
| rs3737482 | 4 | 100349466 | ADH7 |
| rs4147549 | 4 | 100356012 | ADH7 |
| rs993702 | 4 | 108390094 | unknown |
| rs3733633 | 4 | 108575989 | PAPSS1 |
| rs2069762 | 4 | 123377980 | IL2 |
| rs997779 | 4 | 176936038 | GPM6A |
| rs1801394 | 5 | 7870973 | FASTKD3,MTRR |
| rs2972994 | 5 | 42756500 | CCDC152 |
| rs3846662 | 5 | 74651084 | HMGCR |
| rs3733890 | 5 | 78421959 | BHMT |
| rs12653308 | 5 | 82858828 | VCAN |
| rs13356198 | 5 | 129257092 | CHSY3 |
| rs1800925 | 5 | 131992809 | IL13 |
| rs2070874 | 5 | 132009710 | IL4 |
| rs17170899 | 5 | 136376050 | SPOCK1 |
| rs2569190 | 5 | 140012916 | CD14 |
| rs10946398 | 6 | 20661034 | CDKAL1 |
| rs742132 | 6 | 25607571 | LRRC16A |
| rs3757131 | 6 | 25783909 | SLC17A1 |
| rs1179086 | 6 | 25791745 | SLC17A1 |
| rs1041981 | 6 | 31540784 | LTA |
| rs1799964 | 6 | 31542308 | LTA,TNF |
| rs1799724 | 6 | 31542482 | LTA,TNF |
| rs6902123 | 6 | 35330421 | PPARD |
| rs2267668 | 6 | 35377922 | PPARD |
| rs2234693 | 6 | 152163335 | ESR1 |
| rs4880 | 6 | 160113872 | SOD2 |
| rs2981977 | 6 | 167750150 | TTLL2 |
| rs909545 | 6 | 167754661 | TTLL2 |
| rs12528714 | 6 | 167754975 | TTLL2 |
| rs1800796 | 7 | 22766246 | IL6 |
| rs1799884 | 7 | 44229068 | GCK |
| rs3897749 | 7 | 86134197 | unknown |
| rs17776650 | 7 | 95747617 | SLC25A13 |
| rs10246939 | 7 | 141672604 | TAS2R38 |
| rs2070744 | 7 | 150690079 | NOS3 |
| rs2069459 | 7 | 150751590 | CDK5 |
| rs7830759 | 8 | 14308995 | SGCZ |
| rs6530778 | 8 | 14313293 | SGCZ |
| rs328 | 8 | 19819724 | LPL |
| rs331 | 8 | 19820405 | LPL |
| rs4994 | 8 | 37823798 | ADRB3 |
| rs619777 | 8 | 102488478 | unknown |
| rs4355801 | 8 | 119923873 | OPG,TNFRSF11B |
| rs1994276 | 8 | 119951519 | TNFRSF11B |
| rs1799998 | 8 | 143999600 | CYP11B2 |
| rs6474850 | 9 | 14662459 | ZDHHC21 |
| rs2890992 | 9 | 14672267 | ZDHHC21 |
| rs10811661 | 9 | 22134094 | unknown |
| rs12351510 | 9 | 28651898 | LINGO2 |
| rs2230808 | 9 | 107562804 | ABCA1 |
| rs2066715 | 9 | 107588033 | ABCA1 |
| rs2066718 | 9 | 107589255 | ABCA1 |
| rs2740483 | 9 | 107690535 | ABCA1 |
| rs1800976 | 9 | 107690709 | ABCA1 |
| rs7895833 | 10 | 69623057 | SIRT1 |
| rs1467568 | 10 | 69675158 | SIRT1 |
| rs4986893 | 10 | 96540410 | CYP2C19 |
| rs1057910 | 10 | 96741053 | CYP2C9 |
| rs743572 | 10 | 104597152 | CYP17A1 |
| rs11200638 | 10 | 124220544 | HTRA1 |
| rs78013 | 11 | 2819259 | KCNQ1 |
| rs7950226 | 11 | 13318139 | ARNTL |
| rs11022775 | 11 | 13373764 | ARNTL |
| rs2278749 | 11 | 13397878 | ARNTL |
| rs2290035 | 11 | 13407771 | ARNTL |
| rs5215 | 11 | 17408630 | KCNJ11 |
| rs12577551 | 11 | 18809542 | PTPN5 |
| rs1001179 | 11 | 34460231 | CAT |
| rs934178 | 11 | 44585933 | CD82 |
| rs1139971 | 11 | 44640268 | CD82 |
| rs2167079 | 11 | 47270255 | ACP2,NR1H3 |
| rs9943582 | 11 | 57005067 | APLNR |
| rs174570 | 11 | 61597212 | FADS2 |
| rs505802 | 11 | 64357072 | SLC22A12 |
| rs3736228 | 11 | 68201295 | LRP5 |
| rs3793976 | 11 | 88919067 | TYR |
| rs1447352 | 11 | 92722761 | MTNR1B |
| rs550447 | 11 | 94412883 | AMOTL1 |
| rs4753145 | 11 | 94440853 | AMOTL1 |
| rs2266788 | 11 | 116660686 | APOA5,ZNF259 |
| rs6589567 | 11 | 116670676 | APOA5 |
| rs11276 | 12 | 14993439 | ART4 |
| rs7958822 | 12 | 27501106 | ARNTL2 |
| rs4964057 | 12 | 27516842 | ARNTL2 |
| rs10771603 | 12 | 30098545 | TMTC1 |
| rs2228570 | 12 | 48272895 | VDR |
| rs10858915 | 12 | 90051547 | ATP2B1 |
| rs17249754 | 12 | 90060586 | ATP2B2 |
| rs35009941 | 12 | 104682528 | TXNRD1 |
| rs2287161 | 12 | 107381140 | MTERFD3 |
| rs11612727 | 12 | 111314289 | CCDC63 |
| rs6490029 | 12 | 111698457 | CUX2 |
| rs3782886 | 12 | 112110489 | BRAP |
| rs2301756 | 12 | 112890776 | PTPN11 |
| rs10492024 | 12 | 113269412 | RPH3A |
| rs2240193 | 12 | 113335661 | RPH3A |
| rs5888 | 12 | 125284748 | SCARB1 |
| rs3782287 | 12 | 125289265 | SCARB1 |
| rs9542950 | 13 | 72999970 | unknown |
| rs1372000 | 13 | 73369642 | PIBF1 |
| rs1130409 | 14 | 20925154 | APEX1,OSGEP |
| rs2230500 | 14 | 61924239 | PRKCH |
| rs1256049 | 14 | 64724051 | ESR2 |
| rs1950902 | 14 | 64882380 | MTHFD1 |
| rs2236225 | 14 | 64908845 | MTHFD1 |
| rs12148604 | 15 | 51501404 | CYP19A1 |
| rs4441215 | 15 | 51556959 | CYP19A1 |
| rs936306 | 15 | 51579598 | CYP19A1 |
| rs752760 | 15 | 51631479 | CYP19A1 |
| rs1800588 | 15 | 58723675 | LIPC |
| rs6078 | 15 | 58833993 | LIPC |
| rs1048943 | 15 | 75012985 | CYP1A1 |
| rs762551 | 15 | 75041917 | CYP1A2 |
| rs1378942 | 15 | 75077367 | CSK |
| rs34713741 | 15 | 101817876 | SELS |
| rs17822931 | 16 | 48258198 | ABCC11 |
| rs9939609 | 16 | 53820527 | FTO |
| rs243865 | 16 | 55511806 | MMP2 |
| rs2285053 | 16 | 55512377 | MMP2 |
| rs3764261 | 16 | 56993324 | unknown |
| rs1800775 | 16 | 56995236 | CETP |
| rs708272 | 16 | 56996288 | CETP |
| rs5882 | 16 | 57016092 | CETP |
| rs6564961 | 16 | 82079091 | HSD17B2 |
| rs4888202 | 16 | 82128422 | HSD17B2 |
| rs2955160 | 16 | 82131578 | HSD17B2 |
| rs4792149 | 17 | 11460645 | SHISA6 |
| rs11078022 | 17 | 11511457 | DNAH9 |
| rs1979277 | 17 | 18232096 | SHMT1 |
| rs2041306 | 17 | 30665675 | C17orf75 |
| rs2036535 | 17 | 31751013 | ACCN1 |
| rs2676531 | 17 | 40704185 | HSD17B1 |
| rs2333227 | 17 | 56358762 | MPO |
| rs1799752 | 17 | 61565890 | ACE |
| rs12945290 | 17 | 66378997 | ARSG |
| rs3861288 | 18 | 40303754 | RIT2 |
| rs17782313 | 18 | 57851097 | MC4R |
| rs12970134 | 18 | 57884750 | MC4R |
| rs713041 | 19 | 1106615 | GPX4 |
| rs2075710 | 19 | 1106845 | GPX4 |
| rs1862513 | 19 | 7733793 | RETN |
| rs157580 | 19 | 45395266 | TOMM40 |
| rs405509 | 19 | 45408836 | APOE |
| rs429358 | 19 | 45411941 | APOE |
| rs7412 | 19 | 45412079 | APOE |
| rs6039211 | 20 | 8616588 | PLCB1 |
| rs3918242 | 20 | 44635976 | MMP9 |
| rs17576 | 20 | 44640225 | MMP9 |
| rs6024692 | 20 | 54777973 | MC3R |
| rs7121 | 20 | 57478807 | GNAS |
| rs2839519 | 21 | 43867844 | UBASH3A |
| rs234706 | 21 | 44485350 | CBS |
| rs13050924 | 21 | 44769969 | FLJ41733 |
| rs1051266 | 21 | 46957794 | SLC19A1 |
| rs4680 | 22 | 19951271 | COMT |
| rs78649 | 22 | 21220651 | SNAP29 |

“name”, “chr”, “position” and “gene” represent the rs numbers, chromosome numbers, physical positions on each chromosome and gene symbols associated with polymorphisms, respectively. Physical positions were determined using assembly GRCh37 patch release 2.
