# Supplementary material for: Detailed Analysis of Japanese Population Substructure with a Focus on the Southwest Islands of Japan
Source: PLoS One. 2012 Apr 3;7(4):e35000. doi: 10.1371/journal.pone.0035000 (PMC3318002; doi:10.1371/journal.pone.0035000)
Supplement: Table S2 — (DOC) [file pone.0035000.s007.doc]

**Table S2**

| (a) Genetic differentiation among populations in the J-MICC data | | |  |  |  |
| --- | --- | --- | --- | --- | --- |
|  | Tokai-Hokuriku | Kinki | Chugoku-Shikoku | Kyushu | Amami Islands |
| Kanto-Koshinetsu | 0.0003 | 0.0002 | 0.0011 | 0.0002 | 0.0079 |
|  | (0.0001, 0.0005) | (-0.0001, 0.0004) | (0.0003, 0.0019) | (0.0000, 0.0004) | (0.0064, 0.0096) |
| Tokai-Hokuriku |  | 0.0002 | 0.0005 | 0.0002 | 0.0083 |
|  |  | (0.0001, 0.0003) | (-0.0001, 0.0012) | (0.0000, 0.0003) | (0.0068, 0.0101) |
| Kinki |  |  | 0.0008 | 0.0003 | 0.0089 |
|  |  |  | (0.0000, 0.0016) | (0.0002, 0.0005) | (0.0071, 0.0109) |
| Chugoku-Shikoku |  |  |  | 0.0007 | 0.0097 |
|  |  |  |  | (0.0001, 0.0013) | (0.0074, 0.0123) |
| Kyushu |  |  |  |  | 0.0072 |
|  |  |  |  |  | (0.0058, 0.0089) |
| (b) Genetic differentiation between the Amami and the mainland population | | | |  |  |
|  |  |  |  |  |  |
|  | Amami Islands |  |  |  |  |
| Mainland | 0.0080 |  |  |  |  |
|  | (0.0068, 0.0097) |  |  |  |  |

F_ST_ values were estimated as the ratio of sums of variance components in the numerator and denominator, and 95% confidence intervals are computed using 10000 bootstrap resamplings. The mainland population is grouped across all subpopulations in the mainland, *i.e.*, Kanto-Koshinetsu, Tokai-Hokuriku, Kinki, Chugoku-Shikoku, and Kyushu.
