# Supplementary material for: Detailed Analysis of Japanese Population Substructure with a Focus on the Southwest Islands of Japan
Source: PLoS One. 2012 Apr 3;7(4):e35000. doi: 10.1371/journal.pone.0035000 (PMC3318002; doi:10.1371/journal.pone.0035000)
Supplement: Table S3 — (DOC) [file pone.0035000.s008.doc]

**Table S3**

**Genetic differentiation between the mainland population, Amami Islanders and Okinawa Islanders**

|  | Amami Islands | Okinawa Islands (PASNP) |
| --- | --- | --- |
| Mainland | 0.0117 | 0.0193 |
|  | (0.0024, 0.0190) | (-0.0050, 0.0509) |
| Amami Islands |  | 0.0016 |
|  |  | (-0.0055, 0.0107) |

F_ST_ values were estimated as the ratio of sums of variance components in the numerator and denominator, and 95% confidence intervals are computed using 10000 bootstrap resamplings. The mainland population is grouped across all subpopulations in the mainland, *i.e.*, Kanto-Koshinetsu, Tokai-Hokuriku, Kinki, Chugoku-Shikoku, and Kyushu. The genotype data for the Okinawa Islanders were obtained from the HUGO Pan—Asian SNP consortium.
